# Socioeconomic Disparity in Breast Cancer Detection in Hong Kong – A High Income City: Retrospective Epidemiological Study Using the Breast Cancer Registry

**DOI:** 10.1371/journal.pone.0107630

**Published:** 2014-10-01

**Authors:** Josette Sin Yee Chor, Holly Ching Yu Lam, Amy Chan, Hang Mei Lee, Eliza Fok, Sian Griffiths, Polly Cheung

**Affiliations:** 1 Jockey Club School of Public Health and Primary Care, The Chinese University of Hong Kong, Shatin, Hong Kong, Hong Kong; 2 Hong Kong Breast Cancer Foundation, North Point, Hong Kong, Hong Kong; Sudbury Regional Hospital, Canada

## Abstract

**Background:**

It is not known whether socioeconomic disparities affect the detection of breast cancer in Asian countries where the incidence of breast cancer is a rising trend. In this study, we explore the socioeconomic profiles of women and the stage of the disease at the time of diagnosis in breast cancer patients aged 40 or over in Hong Kong.

**Method:**

During the period 2008 to 2011, 5393 breast cancer patients registered with the Hong Kong Breast Cancer Registry. Participants and their clinicians were asked to complete standardised questionnaires including patient socio-demographics, health history and risk factors, the course of the disease, post-treatment physical discomfort and psychosocial impact, follow-up recurrence and survival status.

**Results:**

Monthly household incomes, educational levels and the practice of regular screening are independently associated with the stage of the disease at diagnosis. Higher socioeconomic status and a higher educational level were associated with an earlier stage of the disease at the time of diagnosis. Yearly clinical examinations, ultrasound and mammographic screening every 2 to 3 years were significantly associated with the earlier detection of breast cancer.

**Conclusion:**

There were socioeconomic disparities among Hong Kong women who were found to have breast cancer. Population-based screening policies, including raising awareness among women at risk, should be implemented.

## Introduction

Breast cancer is the commonest cancer among women worldwide and the mortality rate is associated with the stage of the disease at the time of diagnosis [1]. Major socioeconomic disparities in women who were found to have breast cancer have been evident in countries with mainly private health services such as the United States of America or in low and middle income countries [2].

Disparities are mostly seen among different ethnic groups. Black and Hispanic women, as well as foreign immigrants [3]–[7], are more likely to be diagnosed with cancer at a later stage than Caucasians. The disparities among different ethnic groups may be due not only to biological differences in tumours but also to the ease of access to screening programmes [8]. Socioeconomic factors and educational levels contribute to the disparity in the stage of breast cancer at presentation and the subsequent rate of survival [9]–[10].

Very few studies have been conducted in high income countries with national health services [11]. Even less is known about the situation in Asian countries, particularly in Chinese communities such as Hong Kong. Hong Kong's health care system aims to provide equal access for all citizens; its per capita GDP was US$34386 in 2011. However, it has a high Gini coefficient as well as disparities. Despite the overall life expectancy ranking second in the world in 2011, the breast cancer incidence rate has increased over the past two decades, possibly because of the adoption of Western lifestyles. Unlike breast cancer mortality rates in other developed countries, the mortality rate in Hong Kong has remained unchanged. One contributory factor is the lack of population-based screening in Hong Kong. This study also examines whether screening practices are associated with the early detection of breast cancer.

Our study aims to explore the relationship between social inequality and different screening modalities in the detection of breast cancer in Hong Kong women.

## Method

With written consent, newly diagnosed breast cancer patients (including in situ and invasive breast cancers) were registered with the Hong Kong Breast Cancer Registry (HKBCR). HKBCR was established in 2007 by the Hong Kong Breast Cancer Foundation and, using a standardised questionnaire, captures the data of both public and private breast cancer patients attending surgical departments in Hong Kong.

Participants were recruited in both major private and public breast clinics in all 18 districts of Hong Kong. All patients attending these clinics with a confirmed diagnosis of breast cancer were invited to participate in the study. Participants and their clinicians were asked to complete standardised questionnaires to record different aspects of the disease, from patient demographics, health history and risk factors, to extensive data on the course of the disease including the detection and diagnosis modality, symptoms and signs presented, post-treatment physical discomfort and psychosocial impact, follow-up recurrence and survival status.

### Ethics Statement

The study is conducted with the compliance of the declaration of Helsinki. The study was approved by the Research Ethics Committees of Kowloon Central/Kowloon East, Kowloon West, New Territories East, new Territories West, Hong Kong East, and Hong Kong West under the Hospital Authority Hong Kong.

### Statistical analysis

The association of stage of cancer detected at diagnosis with sociodemographic factors and screening practices were determined by using multiple logistic regression. With the use of multiple logistic regressions, covariates and potential confounders can be adjusted and the independent association of individual study factor with cancer stage at diagnosis can be evaluated. Statistical analyses were performed using SPSS 18.0. All tests are two sided and determined to be significant if p≦0.05.

## Results

During the period 2008 to 2011, 5393 breast cancer patients registered with the HKBCR. 2539 (47.1%) were recruited from private clinics/hospitals and 2854 (52.9%) were recruited from public hospitals.

We have included only women aged 40 or over in the analysis. Of the 3469 subjects we recruited with a complete dataset, women aged below 40 comprised only 13.6% (469) of the whole population and their breast cancer presentations were usually more aggressive and had a different natural history than those occurring at an older age. Therefore, they are excluded from our current analysis.

Data of 2987 women aged 40 or over, residents in all 18 districts of Hong Kong, recruited from both public and private hospitals during the period 2008 to 2011 were analyzed. Women were grouped into 2 cancer stages, the early stage (stage 0, I, IIA & IIB) and the late stage (IIIA, IIIB, IIIC & IV), according to AJCC Cancer Staging Classification (7^th^ edition) (see Appendix S1). Among the 2891 subjects with breast cancer, 13.7% (397) were at late stage at the time of diagnosis and 86.3% (2494) were at early stage at the time of diagnosis. Table 1 shows the distribution of sociodemographics and cancer stages.

**Table 1:** Socio-demographics characteristics of the participants.

Factor	n	Percentage (%)
Age		
40–49	1463	49
50–59	1016	34
60–69	339	11.3
70–79	134	4.5
80 or above	35	1.2
Total	2987	100
Educational level		
No schooling/kindergarten	150	5.0
Primary (Primary school)	762	25.5
Secondary (middle school & high school)	1451	48.6
Matriculation/diploma	214	7.2
Undergraduate or above	363	12.2
Total	2940	100
Monthly household income (HK$)		
<10,000	425	14.2
10,000–29,999	783	26.2
30,000 or above	648	21.7
Total	1856	100
Cancer stage at diagnosis		
0	342	11.8
I	920	31.8
IIA	836	28.9
IIB	396	13.7
IIIA	230	8.0
IIIB	29	1.0
IIIC	107	3.7
IV	31	1.1
Total	2891	100

### Association of sociodemographics with stage at diagnosis

Since household monthly income was highly associated with personal education level and residential district (p<0.0005 in Chi-sq. test), the association with household monthly income was evaluated separately with age group, personal education level and residential district. In a multivariate model adjusted for age and residence district, the educational level was found to be an independent associating factor with stage at diagnosis. Women with a secondary school education, and undergraduate or above educational level, had a higher detection rate of early stage cancer compared with those with no education/at the kindergarten educational level as shown in Table 2 (Adjusted OR (95%CI) = 1.696(1.042, 2.759) and 1.873(1.028, 3.411), respectively).

**Table 2:** Association of education level with detection of cancer at early stage.

				95% C.I. for OR	
Factor	Sub-group (n)	*p*-value	OR	Lower	Upper
Education level	No education/kindergarten (136)		1	—	—
	Primary (719)	0.185	1.390	0.854	2.262
	Secondary (1395) *	0.034	1.696	1.042	2.759
	Matriculation/diploma (199)	0.127	1.637	0.870	3.081
	Undergraduate or above (332)*	0.040	1.873	1.028	3.411

Result of logistic regression with age group and residential district adjusted (N = 2781).

*significant association as *p*<0.05.

**Figure 1:**
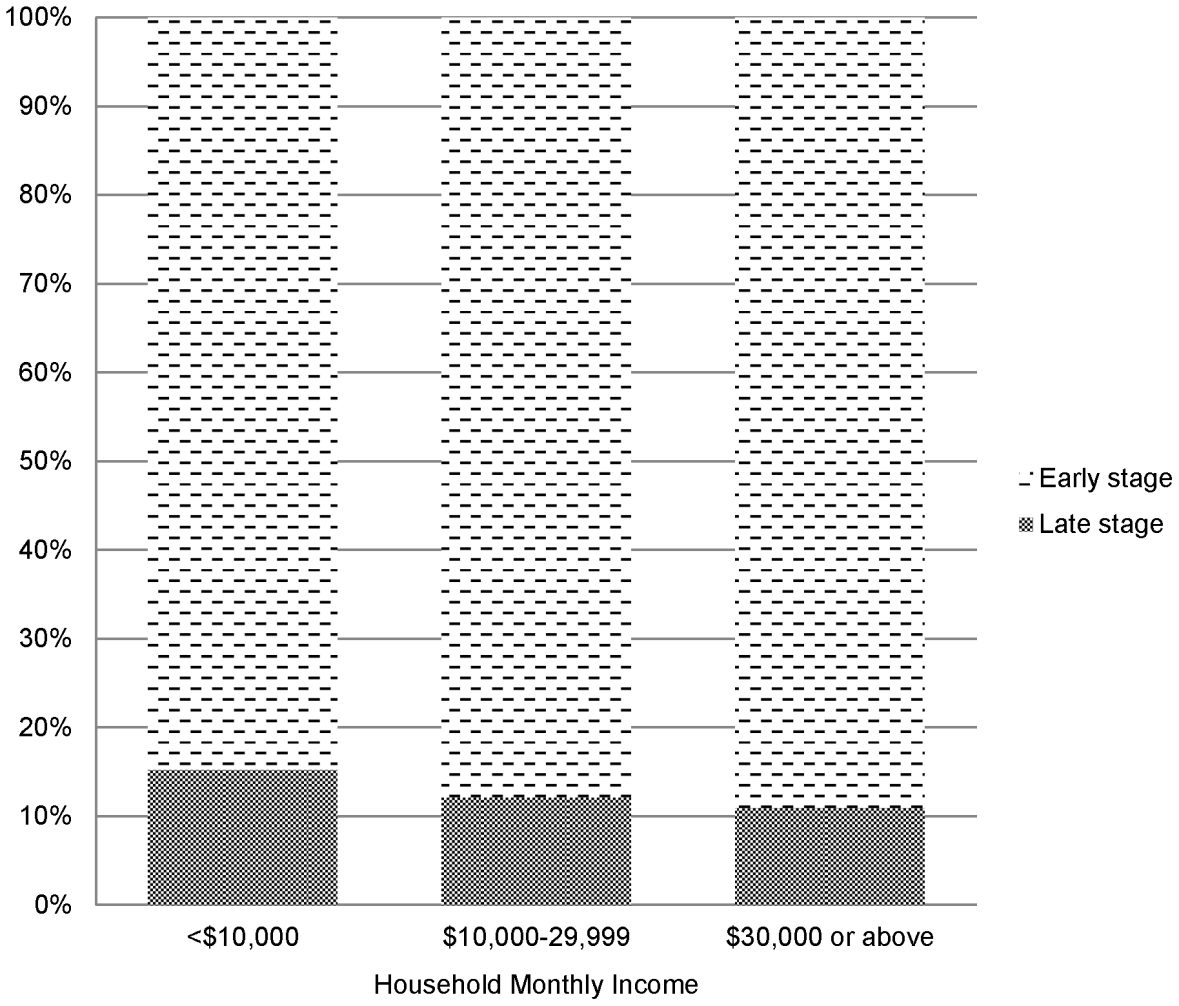
Percentage of early stage cancer detection by household monthly income.

Monthly household income was found to be a significant factor associated with early stage cancer detection (Table 3). Women with a monthly household income of HK$30000 or above had a higher detection rate of early stage cancer (Adjusted OR (95%CI) = 1.453 (1.006, 2.100)) compared with those having a monthly household income of less than HK$10000 (See figure 1). This is probably due to having sufficient means and easy access to a screening service, since it is not provided in the public sector.

**Table 3:** Association of monthly household income with detection of cancer at early stage.

				95% C.I. for OR	
Factor	Sub-group (n)	*p*-value	OR	Lower	Upper
Monthly household income	<HKD10,000 (407)		1	—	—
	HKD10,000-29,999 (765)	0.140	1.299	0.918	1.836
	HKD30,000 or above (627)*	0.047	1.453	1.006	2.100

Result of logistic regression (N = 1799).

*significant association as *p*<0.05.

There is no independent association between age and residence district with the stage at diagnosis.

### Association of the screening practice with stage at diagnosis

Since there was strong association between the practice of performing mammogram and ultrasound (About 80% of subjects performed both screenings at the same frequency, p<0.0005 in Chi-sq. test), to avoid multicollinearity in logistic regression modeling, mammogram and ultrasound were put into different logistic regressions with clinical examination for Odds Ratio calculation (with age-group, monthly household income and personal education level adjusted).

Regular screening practices, including clinical examination, ultrasound examination and mammography, were associated with the stage of the disease at diagnosis. They are all independent predicting factors of the stage of the cancer at presentation (Table 4, 5 and 6).

**Table 4:** Association of the practice of mammogram with detection of cancer at an early stage.

				95% C.I. for OR	
Screening modality	Screening frequency (n)	*p*-value	OR	Lower	Upper
Mammogram	Never (1002)		1	—	—
	Occasionally (191)	0.706	0.917	0.585	1.437
	Yearly (319)*	0.037	1.599	1.028	2.486
	In every 2-3 years (173)*	0.021	2.093	1.116	3.924
	In every>3 years (36)	0.372	1.735	0.517	5.819

Result of logistic regression with age, education level and monthly household income adjusted (N = 1721).

*significant association as *p*<0.05.

**Table 5:** Association of the practice of ultrasound with detection of cancer at early stage.

				95% C.I. for OR	
Screening modality	Screening frequency (n)	*p*-value	OR	Lower	Lower
Ultrasound	Never (1058)		1	—	—
	Occasionally (170)	0.806	1.063	0.651	1.736
	Yearly (273)*	0.029	1.690	1.056	2.705
	In every 2–3 years (94)*	0.028	2.819	1.118	7.107
	In every>3 years (29)	0.307	2.139	0.497	9.197

Result of logistic regression with age, education level and monthly household income adjusted (N = 1624).

*significant association as *p*<0.05.

**Table 6:** Association of the practice of clinical examination with detection of cancer at an early stage.

				95% C.I. for OR	
Screening modality	Screening frequency (n)	*p*-value	OR	Lower	Upper
Clinical examination	Never (591)		1	—	—
	Occasionally (242)	0.766	1.068	0.692	1.648
	Yearly* (689)	0.002	1.789	1.244	2.573
	In every 2–3 years (180)	0.411	1.234	0.747	2.040
	In every>3 years (21)	0.936	1.052	0.299	3.702

Result of logistic regression with age, education level and monthly household income adjusted (N = 1723).

*significant association as *p*<0.05.

Women having a mammogram every year and every 2–3 years were more likely to have cancer detected at an early stage (Adjusted OR(95%CI) = 1.599(1.028,2.486) and 2.093 (1.116,3.924) respectively) than those who had never had a mammogram. There was no significant difference between the interval of screening (1 year versus every 2–3 year). Women who received an ultrasound examination every year and every 2–3 years were more likely to have breast cancer detected at an early stage than those subjects who had never received a USG examination (Adjusted OR (95%CI) = 1.690(1.056,2.705) and 2.819(1.118,7.107) respectively). There was also no significant difference between the interval of screening (1 year versus every 2–3 year). Women receiving a yearly clinical examination were more likely to have cancer detected at an early stage than those subjects who had never had a clinical examination (Adjusted OR(95%CI) = 1.789 (1.244, 2.573)).

**Figure 2:**
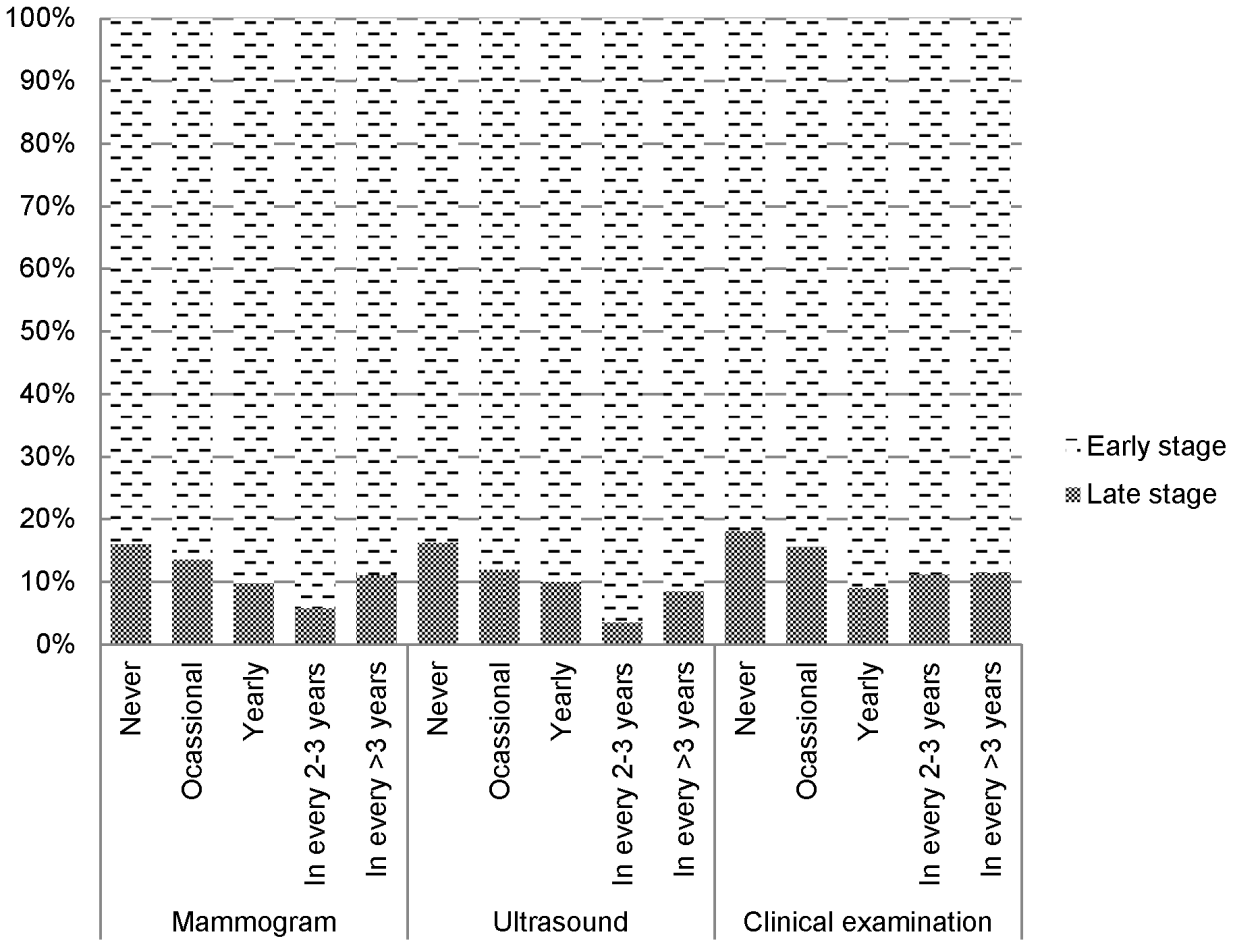
Percentage of early stage cancer detection by practice of screening.

The association with mammogram (every 2–3 years) was marginally significant after adjusted for practice of clinical examination (Table 7). The association with ultrasound (every 2–3 years) and clinical examination persisted even after adjusted by each other (Table 8). The proportions of early stage cancer detection in subjects with different screening practices were shown in figure 2.

**Table 7:** Association of the screening practices (clinical examination and mammogram) with detection of cancer at an early stage.

				95% C.I. for OR	
Screening modality	Screening frequency	*p*-value	OR	Lower	Upper
Clinical Examination	Never (588)		1	—	—
	Occasionally (239)	0.673	1.109	0.687	1.789
	Yearly* (675)	0.019	1.670	1.086	2.569
	In every 2–3 years (176)	0.891	1.039	0.599	1.804
	In every>3 years (21)	0.785	0.825	0.207	3.292
Mammogram	Never (996)		1	—	—
	Occasionally (190)	0.543	0.857	0.522	1.408
	Yearly (309)	0.696	1.110	0.659	1.868
	In every 2–3 years∧ (168)	0.087	1.813	0.917	3.586
	In every>3 years (36)	0.421	1.726	0.457	6.529

Result of logistic regression with age, education level and monthly household income adjusted (N = 1699).

∧ Marginally significant as *0.05<p<0.1*;*significant association as *p*<0.05.

**Table 8:** Association of the screening practices (clinical examination and ultrasound) with detection of cancer at early stage.

				95% C.I. for OR	
Screening modality	Screening frequency	*p*-value	OR	Lower	Upper
Clinical Examination	Never (584)		1	—	—
	Occasionally (224)	0.916	1.026	0.636	1.657
	Yearly (613)*	0.019	1.672	1.089	2.567
	In every 2–3 years (167)	0.974	1.009	0.584	1.744
	In every>3 years (21)	0.624	0.698	0.166	2.931
Ultrasound	Never (1053)		1	—	—
	Occasionally (168)	0.950	1.017	0.598	1.731
	Yearly (257)	0.549	1.181	0.686	2.032
	In every 2–3 years (92)*	0.049	2.653	1.006	6.997
	In every>3 years (29)	0.259	2.576	0.498	13.331

Result of logistic regression with age, educational level and monthly household income adjusted (N = 1609).

*significant association as *p*<0.05.

## Discussion

Late stage breast cancer is associated with higher mortality and morbidity due to the need for more aggressive surgical treatment and a chemotherapy/radiotherapy regime. Our findings agree with previous studies showing that breast cancer mortality was associated with socioeconomic status [12]. One of the possible reasons for the disparity is that there is no population-based screening programme in Hong Kong. Opportunistic mammographic screening is provided in the private sector and by non-profit making organisations in Hong Kong and each cost from US$100 to US$200.

Previous studies have indicated that screen detected tumours were found at earlier stages than symptomatic tumours [13]–[14]. Studies in the United States have highlighted the fact that uninsured women were more likely to have tumours presenting at a larger size and with metastases [15]. Our findings in Hong Kong confirm that socioeconomic factors impact on attendance for screening and, hence, early detection and the possibility of earlier intervention. Since educational levels were also associated with the stage at diagnosis as shown in other countries [16], targeted health education would increase breast awareness as well as screening uptake if services were made accessible to all [17].

Our findings showed that both ultrasound and mammographic screening helped to detect cancers at an earlier stage. The association persisted even after the adjustment by demographics and socioeconomic status. Chinese women have small, high density breasts and, therefore, ultrasound examination may be more beneficial especially for younger women. Future studies evaluating screening methodology in Asian women must take these factors into account. More importantly, since the benefits to women of earlier cancer detection are clearly demonstrated, we believe that the need for a population-wide breast screening programme in Hong Kong should be urgently reviewed.

One limitation of this study is the recall bias of the screening practice in the questionnaire. Moreover, there is no information on the characteristics of the non-participants. On the other hand, this study has captured data of both public and private patients in all 18 districts of Hong Kong. Furthermore, it is a prospective study so data on monthly household incomes, educational levels and screening practices can be captured which are not available in routine data available from the Government database.

## Conclusions

In this study, of Hong Kong women in a high income city, we found that socioeconomic inequalities have an impact on whether or not a woman attends for breast screening and the stage of the breast cancer at presentation.

## Supporting Information

Appendix S1 **AJCC Cancer Staging Classification (7th edition).**
(DOCX)
