# Supplementary material for: Socioeconomic Disparity in Breast Cancer Detection in Hong Kong – A High Income City: Retrospective Epidemiological Study Using the Breast Cancer Registry
Source: PLoS One. 2014 Oct 1;9(10):e107630. doi: 10.1371/journal.pone.0107630 (PMC4182676; doi:10.1371/journal.pone.0107630)
Supplement: Appendix S1 — AJCC Cancer Staging Classification (7th edition). (DOCX) [file pone.0107630.s001.docx]

Appendix

| AJCC Cancer Staging Classification (7^th^ edition) | | | |
| --- | --- | --- | --- |
| Stage | T | N | M |
| 0 | Tis | N0 | M0 |
| IA | T1* | N0 | M0 |
| IB | T0 | N1mi | M0 |
|  | T1* | N1mi | M0 |
| IIA | T0 | N1** | M0 |
|  | T1* | N1** | M0 |
|  | T2 | N0 | M0 |
| IIB | T2 | N1** | M0 |
|  | T3 | N0 | M0 |
| IIIA | T0 | N2 | M0 |
|  | T1* | N2 | M0 |
|  | T2 | N2 | M0 |
|  | T3 | N1 | M0 |
|  | T3 | N2 | M0 |
| IIIB | T4 | N0 | M0 |
|  | T4 | N1 | M0 |
|  | T4 | N2 | M0 |
| IIIC | Any T | N3 | M0 |
| IV | Any T | Any N | M1 |
| * T1 includes T1mi | | | |
| ** T0 and T1 tumour with nodal micrometastases only are excluded from Stage IIA and are classified Stage IB. | | | |
